# Zonulin, a marker of gut permeability, is associated with mortality in a cohort of hospitalised peruvian COVID-19 patients

**DOI:** 10.3389/fcimb.2022.1000291

**Published:** 2022-09-06

**Authors:** Luciano A. Palomino-Kobayashi, Barbara Ymaña, Joaquim Ruiz, Ana Mayanga-Herrera, Manuel F. Ugarte-Gil, Maria J. Pons

**Affiliations:** ^1^ Grupo Enfermedades Infecciosas Emergentes. Universidad Científica del Sur, Lima, Peru; ^2^ Laboratorio de Cultivo Celular e Inmunología, Universidad Científica del Sur, Lima, Peru; ^3^ Grupo Peruano de Estudio de Enfermedades Autoinmunes Sistémicas, Universidad Científica del Sur, Lima, Peru; ^4^ Hospital Nacional Guillermo Almenara Irigoyen, EsSalud, Lima, Peru

**Keywords:** microbial translocation, zonulin, COVID-19, biomarker, ELISA, Peru

## Abstract

Zonulin has previously been related to intestinal permeability in various inflammatory diseases, and more recently to the physiopathology of severe COVID-19 infections. We analysed serum samples from a previous study of a Peruvian cohort of hospitalised COVID-19 patients, for the quantification of zonulin by sandwich ELISA. Comparisons with clinical data, haematological and biochemical parameters and cytokine/chemokine levels were made. We found higher baseline zonulin levels in deceased patients, and zonulin was associated with fatal outcome in multivariable analyses, even after adjustment for age, gender, and obesity. There were also positive correlations between zonulin, creatinine, D-dimer values and prothrombin time, while inverse correlations were found for Sa/FiO_2_ ratio and CCL5 (RANTES). Further longitudinal studies are recommended to analyse the variation of zonulin levels over time as well as their relationship with long-COVID.

## Introduction

Severe COVID-19 infections are characterised by systemic inflammation, acute respiratory distress syndrome (ARDS) and multi-organic system failure in adults (Kim et al., 2021). Severe Acute Respiratory Syndrome Coronavirus 2 (SARS-CoV-2) binds principally to receptor angiotensin-converting enzyme 2 (ACE2) to enter many different host cells such as type II pneumocytes in the lung, myocardial cells, neurons and glia, endothelial cells, intestinal enterocytes and others (Lamers et al., 2020; Beyerstedt et al., 2021). Particularly, intestinal enterocytes form a cell layer that is part of the mucosal defence system and are held together by tight junctions. Disruption of this layer (increased gut permeability) translates into translocation of bacterial components from the gut into the bloodstream, contributing to a further increase in systemic inflammation (Dinh et al., 2015).

As reported in other viral infections such as dengue fever (Chancharoenthana et al., 2021) or human immunodeficiency virus (Marchetti et al., 2013), microbial translocation may be caused by direct enterocyte infection from the virus or because of cytokine-mediated alterations and gut dysbiosis. Previous studies (Yonker et al, 2021; Giron et al., 2021; Oliva et al., 2021) have linked higher intestinal translocation with severe cases of COVID-19, describing a higher abundance of bacterial components in the bloodstream compared to milder cases. It has been suggested that microbial translocation, intestinal dysbiosis and underlying inflammatory processes might have a synergistic effect on systemic inflammation and high cytokine levels described in severe COVID-19 infections (Cardinale et al., 2020; Vignesh et al., 2020; Uzzan et al., 2020; Mashaqi et al., 2022).

Zonulin (pre-Haptoglobin 2) is a protein of ~ 47 kDa, which is detectable in its uncleaved form in human serum (Fasano, 2011) and is highly correlated with bacterial DNAemia (Gargari et al., 2021). It is a physiological mediator that reversibly regulates intestinal permeability by modulation of intercellular tight junctions (Tripathi et al., 2009) and is considered a biomarker of impaired gut barrier function (Fasano, 2011). In this sense, it has been hypothesised that the spike protein of SARS-CoV-2 induces overexpression of zonulin, which is related to an increase in intestinal permeability (Llorens et al., 2021). Moreover, zonulin levels have been reported to be higher in patients who died of severe COVID-19 (Giron et al., 2021). These data suggest that SARS-CoV-2 might facilitate passage of intestinal contents into circulation and a higher inflammatory state at intestinal level that could fuel general systemic inflammation. Thus, zonulin might be also an indirect marker of microbial translocation and could be associated with worse outcomes in COVID-19.

Inflammatory responses to viral infection are also influenced by genetic background, for example individual genetic variability of pro-inflammatory cytokines is related to severity in COVID-19 (Vakil et al., 2022). Moreover, population-associated variation of responses to viral infections is differentiated by genetic ancestry (Randolph et al., 2021). The few published studies on serum zonulin levels and COVID-19 come from cohorts/case series of United States of America (Giron et al., 2021; Josyabhatla et al., 2021; Yonker et al., 2022), Italy (Oliva et al., 2021) and Turkey (Kılıç et al., in press) only. Therefore, data from studies with subjects from a different genetic and geographic background are important for external validity issues.

For the reasons mentioned above, the present study aimed to identify the association between microbial translocation estimated by serum zonulin and patient outcome in a cohort of moderate and severe COVID-19 hospitalised patients from Lima, Peru. Additionally, correlations were established amongst cytokine/chemokine values, routine clinical parameters, and serum zonulin levels.

## Materials and methods

### Study population and assessment of disease severity

The present study used samples from a cohort of hospitalised COVID-19 patients from *Hospital Nacional Guillermo Almenara Irigoyen* (Pons et al., 2021). A total of 60 patients were classified according to severity: 19 moderate, 26 severe and 15 deaths (initially classified as moderate or severe). All deaths occurred during hospitalisation. The COVID-19 cases were confirmed by quantitative polymerase chain reaction (qPCR) or antibody testing. The follow-up period was from August to October 2020, which was within the context of the first wave of COVID-19 in Peru. Therefore, no patient had received vaccination for COVID-19.

The definition of moderate infection was according to the Berlin 2012 definition of Acute Respiratory Distress Syndrome (ARDS) (ARDS Definition Task Force, 2012), while severe infection was defined whenever one or more of the following states developed: Horowitz index (PaO_2_/FiO_2_) < 100, oxygen saturation to fraction of inspired oxygen ratio (SpO_2_/FiO_2_, henceforth abbreviated as Sa/FiO_2_) < 89, need for mechanical ventilation due to respiratory failure or systemic organ failure requiring admission to the intensive care unit.

### Sample conditions

Serum samples from the original study were conserved at -80°C in the laboratories of the *Universidad Científica del Sur*.

### Clinical data and cytokine levels

Data pertaining to age, gender, date of hospital admission, date of hospital discharge/death, date of sample according to hospital admission, comorbidities such as obesity, arterial hypertension, diabetes, chronic obstructive pulmonary disease (COPD) and any other underlying conditions, pharmacological treatment received, haemoglobin, platelets, white blood cell (WBC) counts and Sa/FiO_2_ were obtained from the corresponding medical records. All privileged identifiable information of the patients was excluded from the dataset generated and the samples were codified previously to ensure confidentiality.

The cytokine and chemokine concentrations of the serum samples were obtained from the data previously reported in the study by Pons et al. (Pons et al., 2021), in which a commercial kit of Luminex MAGPIX technology (Merck KGaA, Darmstadt, Germany) was used for quantification.

### Estimation of microbial translocation by zonulin quantification

Zonulin (ng/mL) was quantified from the serum samples by sandwich ELISA, using the CUSABIO Human Zonulin ELISA Kit (CUSABIO TECHNOLOGY LLC, Wuhan, China) according to the manufacturer’s instructions. Briefly, 100 µL of standards and samples were added per well in the coated microplate and incubated for two hours at 37°C. Subsequently, 100 µL of biotin-antibody solution was added and incubated for one hour at 37°C. Afterwards, the liquid was aspirated, and manual washing was done three times with the provided wash buffer. Then 100 µL of horseradish peroxidase-avidin solution was added per well and incubated for one hour at 37°C, and manual washing was done thereafter. Finally, 90 µL of 3,3’,5,5’-Tetramethylbenzidine (TMB) substrate was added per well and incubated for 30 minutes at 37°C. Immediately after, 50 µL of stop solution was added per well and absorbance reading was done at 450 nm (with correction at 540 nm) in a Synergy LX Multi-Mode Reader (Biotek, USA). The samples and standards were run in duplicate.

### Statistical analysis

Descriptive analysis was performed using absolute and relative frequencies for summarising categorical variables while median and interquartile range (IQR) were used for numerical variables, unless specified otherwise.

Bivariate analyses were made for comparisons between outcomes (survived vs. deceased) and categorical variables using the Chi-squared or Fisher exact test, while the Mann-Whitney U test was used for comparisons between outcomes and numerical variables. Correlations among WBC counts, biochemical markers, cytokines, chemokines and zonulin levels were normalised using base 10 log transformation and comparisons were made using Spearman tests.

Crude and adjusted multivariable analyses of potential prognostic factors of death were performed using logistic regression with clinical variables, cytokine and zonulin levels at baseline (from first serum sample available since hospital admission) and odds ratios (OR) were obtained. Survival analyses were done using Cox regression, and crude and adjusted hazard ratios (HR) were estimated accordingly. Significant variables in bivariate analysis were employed for the regression analyses. Epidemiological criteria for adjustment of confounding and the use of variables with less than 20% data loss were employed to decide which adjusted model best fitted the data.

For the interpretation of the zonulin results, samples with a zonulin concentration above the upper limit of the standard (40 ng/mL) were assigned this value. Values below 0.625 ng/mL were used as their actual values for the analyses.

The analyses were conducted using STATA 16.1 SE Edition (StataCorp, TX) and graphics were constructed using R version 4.1.2. 95% Confidence intervals were calculated for OR and HR. Statistical significance was established with α=0.05.

## Results

### Baseline clinical characteristics of the hospitalised cohort

Sixty hospitalised COVID-19 patients were included: 45 patients (75%) survived, and 15 (25%) patients died during the follow-up period. The distribution for case severity was as follows: 39 patients (65%) were classified as severe and 21 (35%) as moderate according to the given case definition and progression during the follow-up period. Severity was associated with outcome (p=0.042). The median age was similar in both the survived and deceased groups (55 and 55 years, respectively) and there was no significant difference in age (p=0.694) or sex (p=0.678) between the two groups. The median (IQR) time from admission to first serum sample were 1(1.5) and 1(5) days in the survived and deceased groups respectively (p=0.621). Time from admission to hospital discharge/death (time of hospitalisation) and time from first serum sample obtained to hospital discharge/death were also similar between the aforementioned groups (p=0.978 and p=0.461, respectively) (**Table 1**).

**Table 1:** Characteristics of the study cohort by outcome at baseline.

	Survived	Deceased	p-value
Overall, n=60 (%)	45 (75.00)	15 (25.00)	
Age (years)	55 (19)	55 (25)	0.6943
Sex			
Male (%)	39 (86.67)	12 (80.00)	0.6780
Female (%)	6 (13.33)	3 (20.00)	
Severity			
Moderate (%)	19 (42.22)	2 (13.33)	**0.0420**
Severe(%)	26 (57.78)	13 (86.67)	
Time from admission to hospital discharge/death (days)	11 (8)	12.5 (12)	0.978
Time from admission to first sample (days)	1 (1.5)	1 (5)	0.621
Time from first sample to hospital discharge/death (days)	9(8.5)	8(14)	0.461
Presence of Comorbidities, n (%)			
None	23 (51.11)	3 (20.00)	**0.035**
Obesity	9 (20.00)	9 (64.29)	**0.006**
Diabetes	5 (11.11)	2 (14.29)	0.666
High Blood Pressure	8 (17.78)	5 (35.71)	0.266
CKD	1 (2.22)	0 (0.00)	1.000
COPD	1 (2.22)	2 (14.29)	0.137
CVD	0 (0.00)	1 (7.14)	0.237
Gout	1 (2.22)	0 (0.00)	1.000
Other	3 (6.67)	0 (0.00)	0.566
Haemoglobin, white blood cell count and other biomarkers			
Haemoglobin (g/dL), mean (SD)	14.38 (1.52)	13.19 (1.93)	**0.0176**
Leucocytes (10^9^/L)	9 (6.87)	12.485 (8.17)	0.2142
Band neutrophils (%)	2 (3)	2 (2)	0.6491
Segmented neutrophils (%)	83 (12)	88 (7)	**0.0060**
Lymphocytes (%)	10 (10)	6 (4)	**0.0045**
Total neutrophils (10^9^/L)	7.56 (7.32)	10.82 (8.26)	0.2307
Total lymphocytes (10^9^/L)	0.94 (0.77)	0.59 (0.33)	**0.0046**
Platelets (count per µL)	345000 (147000)	274500(45000)	0.1850
CRP (mg/mL)	61.3 (163.8)	168.7 (122.8)	0.1701
D-dimer (µg/mL)	0.58 (0.51)	0.85 (1.86)	0.1425
Ferritin (µg/L)	789.7 (679.5)	1261.1 (838)	0.0536
Creatinine (mg/dL)	0.7 (0.2)	0.8 (0.48)	0.4152
PT (s)	11.17 (0.89)	11.57 (1.31)	0.1627
APTT (s)	30.87 (5.15)	32.65 (4.22)	0.1782
Sa/FiO_2_	240 (162.63)	108.73 (35.40)	**0.0007**

Values of continuous variables are reported as median (interquartile range), unless stated otherwise. CKD, Chronic Kidney Disease; COPD, Chronic Obstructive Pulmonary Disease; CVD, Cardiovascular disease; CRP, CReactive Protein; PT, Prothrombin Time; APTT, Activated Partial Thromboplastin Time; SaFiO_2_, Oxygen saturation/Fraction of inspired oxygen.

Some comorbidities such as obesity, diabetes, high blood pressure and others were present (**Table 1**). In the deceased group, 12 patients (80%) had comorbidities compared to 22 (48.9%) in the survivor group, and this difference was significant (p=0.035). The only comorbidity that was associated with outcome was obesity (p=0.006). Corticosteroids were employed as part of the treatment for all the patients in this cohort.

### White blood cell counts, biochemical markers, and cytokine/chemokine levels at baseline

Haemoglobin levels at baseline were lower in the deceased, with a mean (standard deviation) of 13.19 (1.93) g/dL (p=0.018). Regarding WBC counts, segmented neutrophil counts were higher (p=0.006) and lymphocyte counts were lower in the deceased group (p=0.004) with no significant differences being found for the remaining WBC counts in the two groups.

In relation to some biochemical markers such as C-reactive protein, D-dimer and ferritin, there were no significant differences when comparisons were made by outcome (**Table 1**). However, the Sa/FiO_2_ ratio was lower in the deceased group, with a median (IQR) of 108.73 (35.40) (p=0.0007).

The cytokines inteleukin-6 (IL-6) (p=0.029), granulocyte-macrophage colony-stimulating factor (GMCSF) (p=0.005), IL-15 (p=0.046), IL-2 (p=0.027), IL-8 (p=0.0004), IL-1a (p=0.032), chemokines macrophage inflammatory protein-alpha (MIPα) (p=0.008) and monocyte chemoattractant protein-1 (MCP-1) (p=0.001) showed significant differences according to outcome (**Table 2**), with all being elevated in the deceased group.

**Table 2:** Zonulin, cytokines and chemokines values in serum at baseline by outcome.

	Survived	Deceased	p-value
**Overall**, n=60 (%)	45 (75.00)	15 (25.00)	
Zonulin (ng/mL)	1.0029 (0.8887)	3.2644 (4.7096)	0.0002
			
**Overall**, n=42 (%)	37 (88.1)	5 (11.90)	
IL-1β (pg/mL)	10 (1.5)	11 (2)	0.1194
GCSF (pg/mL)	11.5 (3)	12 (3)	0.3083
IL-10 (pg/mL)	19.5 (11)	49.5 (54)	0.1246
IL-13 (pg/mL)	8 (2)	8(2)	0.2062
IL-6 (pg/mL)	21 (38)	55 (130)	**0.0295**
IL-12 (pg/mL)	13 (3)	15 (4)	0.5556
IL-17A (pg/mL)	14 (2)	14 (3)	0.4770
GMCSF (pg/mL)	11 (2.5)	14 (4)	**0.0059**
IL-15 (pg/mL)	14 (3)	18 (8)	**0.0456**
EGF (pg/mL)	43 (64)	25 (135)	0.8612
IL-5 (pg/mL)	11 (1)	12 (1.5)	0.3165
VEGF (pg/mL)	20 (15.5)	17 (110)	0.6688
IFN-γ (pg/mL)	10 (3)	11 (2)	0.7092
IFN-α (pg/mL)	10 (1)	11 (2)	0.2605
IL1-RA (pg/mL)	12 (4.5)	18 (33.5)	0.0928
TNF-α (pg/mL)	27.5 (10)	50.5 (44)	0.0566
IL-2 (pg/mL)	13 (1)	15 (2)	**0.0275**
IL-7 (pg/mL)	13 (4)	15 (3)	0.3299
IL-4 (pg/mL)	8 (2)	10 (2)	0.1539
IL-8 (pg/mL)	66 (28.5)	174 (258.5)	**0.0004**
IL-12p70 (pg/mL)	9 (1)	10 (2)	0.1730
IL-1a (pg/mL)	14 (4)	21 (5)	0.0318
IL-3 (pg/mL)	8 (1)	8 (1)	0.1627
TNF-β (pg/mL)	10 (2)	10 (2)	0.9191
			
**Overall**, n=42 (%)	37 (88.1)	5 (11.90)	–
RANTES (pg/mL)	11089 (3360)	10507 (4039.5)	0.6000
Eotaxin (pg/mL)	98 (69)	134 (123.5)	0.8308
MIP-1α (pg/mL)	15.5 (5.5)	22 (23.5)	**0.0081**
MIP-1β (pg/mL)	118 (99)	173 (589.5)	0.2211
MCP-1 (pg/mL)	1620 (1938)	9113 (1550)	**0.0008**
IP-10 (pg/mL)	3501.5 (5094)	6720 (4416)	0.1563

Values of continuous variables are reported as median (interquartile range).

### Correlations of zonulin versus haematological/biochemical markers and cytokines/chemokines at baseline

**Figure 1:**
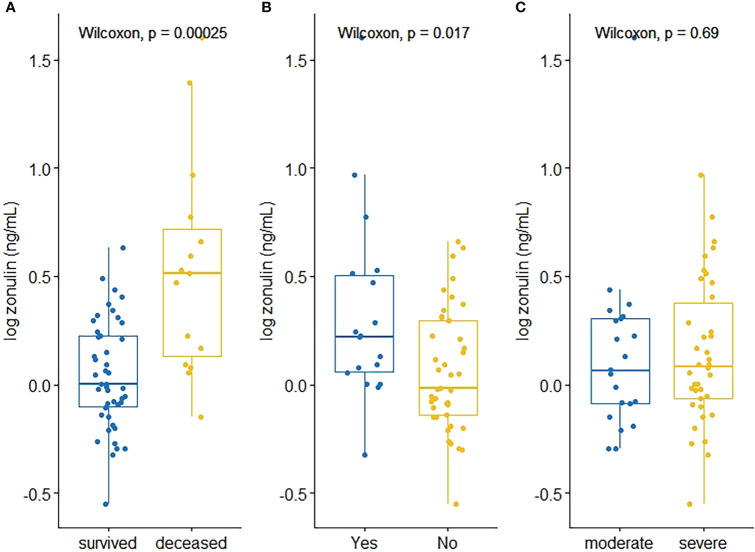
Scatterplots depicting significant correlations between zonulin and D-dimer **(A)**, Creatinine **(B)**, Prothrombin Time **(C)**, Sa/FiO_2_ **(D)** and RANTES **(E)**.

Zonulin levels were positively correlated with creatinine (ρ=0.288, p=0.035) D-dimer values (ρ=0.441, p=0.002), whereas prothrombin time (ρ=0.344, p=0.011) and Sa/FiO_2_ ratio (ρ=-0.4764, p=0.0001) were found to be negatively correlated with zonulin (**Table 3**, **Figure 1**).

**Table 3:** Correlations between zonulin levels and haematological / biochemical markers and cytokines/chemokines at baseline.

	Spearman’s rho	p-value
Haematological/biochemical marker		
Haemoglobin	-0.2009	0.1305
Leukocytes	0.0027	0.9840
Band neutrophils	0.1993	0.2824
Segmented neutrophils	0.0933	0.4859
Lymphocytes	-0.0374	0.7803
Platelets	-0.0852	0.5287
CRP	0.0114	0.9354
D-dimer	0.4415	**0.0024**
Ferritin	0.1846	0.1654
Creatinine	0.2880	**0.0347**
PT	0.3441	**0.0108**
APTT	0.1311	0.3447
Sa/FiO_2_	-0.4764	**0.0001**
Cytokines		
IL-1β	-0.1077	0.4971
GCSF	-0.1817	0.2494
IL-10	0.0117	0.9413
IL-13	-0.0113	0.9434
IL-6	-0.0946	0.5514
IL-12	0.9238	
IL-17A	0.1177	0.4579
GMCSF	0.0231	0.8845
IL-15	0.1361	0.3900
EGF	-0.0478	0.7636
IL-5	-0.1591	0.3143
VEGF	0.1512	0.3392
IFN-γ	-0.0474	0.7657
IFN-α	-0.0334	0.8336
IL1-RA	-0.0612	0.7002
TNF-α	0.2342	0.1354
IL-2	0.0181	0.9092
IL-7	-0.2031	0.1970
IL-4	-0.1908	0.2262
IL-8	0.1990	0.2065
IL-12p70	0.0738	0.6421
IL-1a	-0.0106	0.9467
IL-3	-0.0859	0.5885
TNF-β	-0.1670	0.2905
Chemokines		
RANTES	-0.5023	**0.0007**
Eotaxin	0.1928	0.2212
MIP-1α	-0.0400	0.8016
MIP-1β	0.2032	0.1968
MCP-1	0.2268	0.1486
IP-10	0.0891	0.5745

Related to cytokines and chemokines, RANTES was the only chemokine negatively correlated with zonulin levels (ρ=-0.5023, p=0.007). None of the cytokines were significantly correlated with zonulin levels (**Table 3**, **Figure 1**).

### Zonulin levels at baseline associated with outcome in the bivariate and multivariable analyses

**Figure 2:**
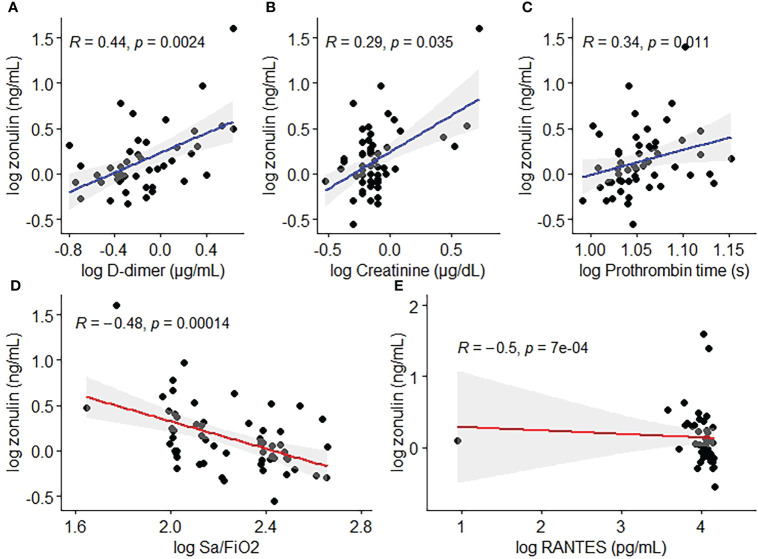
Zonulin concentrations by **(A)** outcome, **(B)** obesity and **(C)** disease severity.

The median (IQR) level of zonulin in the deceased group was 3.264 (4.7096) ng/mL (min 0.7099 ng/mL – max 40 ng/mL, **Table 2**) showing a significant association with outcome (p=0.0002, **Figure 2**) and obesity (p=0.017, **Figure 2**) in the bivariate analysis. Notwithstanding, zonulin was not found to be associated with disease severity (p=0.69, **Figure 2**) in the same analysis.

Several factors in logistic regression, such as obesity (OR 7.200, p=0.003), segmented neutrophils (OR 1.145, p=0.015), GMCSF (OR 2.220, p=0.015), IL-15 (OR 1.332, p=0.031), IL-2 (OR 3.089, p=0.025), and zonulin levels (OR 2.573, p=0.003), presented significant OR values greater than 1 in the crude analysis, which indicate an association with higher odds of mortality. In contrast, haemoglobin (OR 0.624, p=0.030), lymphocytes (OR 0.821, p=0.018) and Sa/FiO_2_ (OR 0.985, p=0.007) had significant ORs less than 1. (**Table 4**).

**Table 4:** Crude and adjusted* logistic regression analysis for predictability of death according to different factors at baseline.

Variables	Crude	Adjusted
	OR (95% CI) p value	OR (95% CI) p value
**Severity**		
Severe	4.750 (0.957-23.572) p=0.057	3.518 (0.593-20.874) p=0.166
Moderate*		
**Obesity**		
Yes	7.200 (1.933-26.812) **p=0.003**	8.103 (2.067-31.768) **p=0.003**
No*		
**Haemoglobin**	0.624 (0.408-0.955)**p=0.030**	0.703 (0.416-1.186) p=0.187
**Seg Neutrophils**	1.145 (1.027-1.278) **p=0.015**	1.303 (1.064-1.596) **p=0.010**
**Lymphocytes**	0.821 (0.696-0.967) **p=0.018**	0.726 (0.563-0.936) **p=0.013**
**SaFiO_2_**	0.985 (0.974-0.996) **p=0.007**	0.987 (0.976-0.998) **p=0.025**
**IL-6**	1.012 (0.996-1.028) p=0.157	1.011 (0.994-1.030) p=0.212
**GMCSF**	2.220 (1.166-4.224) **p=0.015**	2.222 (0.977-5.050) p=0.057
**IL-15**	1.332 (1.026-1.730) **p=0.031**	1.233 (0.939-1.620) p=0.132
**IL-2**	3.089 (1.153-8.274) **p=0.025**	2.555 (0.839-7.778) p=0.099
**IL-8**	1.121 (0.977-1.287) p=0.103	($)
**IL1a**	1.056 (0.953-1.169) p=0.297	1.026 (0.920-1.146) p=0.641
**MIPα**	1.374 (0.933-2.022) p=0.108	1.397 (0.931-2.097) p=0.106
**MCP-1**	1.001 (1.000-1.001) **p=0.010**	1.002 (0.999-1.004) p=0.112
**Zonulin**	2.573 (1.371-4.828) **p=0.003**	2.666 (1.310-5.426) **p=0.007**

*Adjustment for age, gender, and obesity. Obesity was adjusted for age and gender only. Cytokines were adjusted for age and obesity only due to collinearity issues with gender. ($): Value could not be computed as log likelihood = 0.

Notwithstanding, in the adjusted regression, only obesity (only adjusted for age and gender, OR 8.103 p=0.003), segmented neutrophils (OR 1.303, p=0.010), lymphocytes (OR 0.726, p=0.013), Sa/FiO_2_ (OR 0.987, p=0.025) and zonulin levels (OR 2.666, p=0.007) remained significant after adjustment for age, gender, and obesity (**Table 4**).

### Cox regression analysis

Cox regression was performed as a survival analysis with the same predictors as logistic regression, considering the period between date of hospital admission to date of hospital discharge/death (days). In the crude Cox regression only obesity (HR 3.923, p=0.015), Sa/FiO_2_ (HR 0.987, p=0.032), GMCSF (HR 1.975, p=0.034), IL-15 (HR 1.252, p=0.023) and zonulin levels (HR 1.106, p=0.001) were found to be significant (**Table 5**).

**Table 5:** Crude and adjusted* Cox regression of different factors for survival analysis.

Variables	Crude	Adjusted
	**HR (95% CI) p value**	**HR (95% CI) p value**
**Severity**		
Severe	3.436 (0.759-15.566) p=0.109	4.282 (0.796-23.019) p=0.090
Moderate*		
**Obesity**		
Yes	3.923 (1.306-11.783) **p=0.015**	5.888 (1.646-21.068) **p=0.006**
No*		
**Haemoglobin**	0.669 (0.444-1.008) p=0.055	0.770 (0.480-1.235) p=0.278
**Seg Neutrophils**	1.083 (0.980-1.197) p=0.118	1.215 (1.028-1.437) **p=0.023**
**Lymphocytes**	0.874 (0.748-1.020) p=0.087	0.764 (0.623-0.938) **p=0.010**
**SaFiO**_2_	0.987 (0.975-0.999) **p=0.032**	0.986 (0.974-0.998) **p=0.023**
**IL-6**	1.010 (0.997-1.024) p=0.138	1.013 (0.997-1.029) p=0.122
**GMCSF**	1.975 (1.051-3.711) **p=0.034**	2.145 (0.889-5.115) p=0.085
**IL-15**	1.252 (1.032-1.518) **p=0.023**	1.218 (0.987-1.502) p=0.066
**IL-2**	3.080 (0.910-10.427) p=0.070	4.018 (1.001-16.132) **p=0.050**
**IL-8**	1.059 (0.997-1.124) p=0.061	1.080 (0.975-1.196) p=0.142
**IL1a**	1.026 (0.936-1.124) p=0.590	1.011(0.913-1.120) p=0.827
**MIPα**	1.969 (0.917-4.228) p=0.082	2.671 (0.781-9.129) p=0.117
**MCP-1**	1.001 (1.000-1.001) **p=0.020**	1.001 (0.999-1.002) p=0.110
**Zonulin**	1.106 (1.043-1.173) **p=0.001**	1.109 (1.038-1.185) **p=0.002**

*Adjustment for age, gender, and obesity. Obesity was adjusted for age and gender only. Zonulin values were not categorised for the analyses made herein.

As for the case of the adjusted Cox regression (adjustment for age, gender and obesity, obesity was adjusted for age and gender only), the same variables remained significant except GMCSF and IL-15 (**Table 5**). Moreover, segmented neutrophils (HR 1.215, p=0.023), lymphocytes (HR 0.764, p=0.010) and IL-2 (HR 4.018, p=0.050) were not significant in the crude Cox regression analysis but were significant in the adjusted analysis.

## Discussion

Even with readily access to vaccines, COVID-19 is still a threat to public health worldwide with many aspects of its pathophysiology, as well as its consequences after clinical improvement of the infection, requiring study. The present study provides knowledge on the detection of zonulin in COVID-19 patients and some risk factors for death by COVID-19. Serum zonulin levels in a Peruvian cohort of hospitalised COVID-19 patients were measured, and an association was found with mortal outcome (both in bivariate and multivariable analyses, even after adjustment for age, gender, and obesity).

Based on the clinical-epidemiological characteristics of this cohort, the relationship of obesity and fatal outcome in COVID-19 patients has previously been described (Pons et al., 2021). This finding is in agreement with other studies reporting obesity as a strong predictor of death in severe COVID-19 infections (Cai et al., 2021; Dessie and Zewotir, 2021; Yang et al., 2021), as it involves both impaired cellular immunity and an amplification of the underlying obesity-related inflammatory status together with the acute inflammation generated by SARS-CoV-2 infection (Mohammad et al., 2021).

In addition, a lower Sa/FiO_2_ ratio and a decreased lymphocyte count were associated with death by COVID-19 (Henry et al., 2020; Huang and Pranata, 2020; Booth et al., 2021; Pons et al., 2021; Gopalan et al., 2022; Soto et al., 2022). It should be noted that lymphopenia might occur due to consequent apoptosis of activated CD4+T cells by SARS-CoV-2 (Shen et al., 2022), thereby inducing immune suppression.

Although there are few reports about the role of zonulin in COVID-19 infections, Giron et al. (Giron et al., 2021) also reported high zonulin levels in the deceased group, similar to our results. In another study, Oliva et al. (Oliva et al., 2021) found median zonulin values similar to those in our study on comparing COVID-19 patients and matched healthy controls. In addition, it is important to highlight the role of zonulin in children with multisystem inflammatory syndrome (MIS), related to the persistence of the virus in the gastrointestinal tract (Kılıç et al., in press; Josyabhatla et al., 2021). Therefore, zonulin might be associated with a translocation of viral proteins into the bloodstream, contributing to increased inflammation.

Before COVID-19, the zonulin-dependent permeability of intestinal tight junctions had been implicated in chronic inflammatory diseases such as celiac disease, inflammatory bowel disease, type I diabetes (Fasano, 2011), as well as obesity, septicaemia, environmental enteropathy, necrotizing enterocolitis, among others (Sturgeon and Fasano, 2016). In these scenarios, the release of zonulin and loss of gut barrier function are followed by microbial translocation, which triggers immune responses and the production of pro-inflammatory cytokines that exacerbate gut permeability in a vicious cycle (Fasano, 2020). Apart from the intestinal system, overexpression of zonulin has also been reported in other organs such as the lungs and brain (Skardelly et al., 2009; Rittirsch et al., 2013).

Related to COVID-19 infection, SARS-CoV-2 affects the gastrointestinal tract as evidenced by gastrointestinal symptoms and detection of the virus in faeces (Groff et al., 2021). Additionally, viral antigens have been found in intestinal enterocytes after resolution of clinical illness in adults (Gaebler et al., 2021) and zonulin hyper permeability of the mucosal barrier coincides with SARS-CoV-2 antigenemia (Yonker et al., 2021). It has previously been speculated that zonulin participates in a complex mechanism linking the wide variety of symptoms associated with SARS-CoV-2 infection, especially involving the digestive system and the nervous system (Llorens et al., 2021). Specifically, disruption of the intestinal barrier with subsequent microbial translocation could drive inflammatory activation in severe COVID-19 (Giron et al., 2021) with positive feedback of pro-inflammatory cytokines, with the amplified systemic response being stronger in patients with comorbidities (Trottein and Sokol, 2020). Given that our results suggest that zonulin is associated with death, even when adjusted for obesity, this is consistent with the above hypothesis.

Regarding correlations, zonulin was found to be directly correlated with creatinine, D-dimer values, and prothrombin time, all of which have previously been associated with poorer outcome in hospitalised COVID-19 patients (Malik et al., 2021) and inversely correlated with CCL5/RANTES and Sa/FiO_2_. As for the chemokine CCL5 (RANTES), elevated values have been found in moderate but not severe COVID-19 cases and is considered as a prognostic factor for survival (Zhao et al., 2020). It should be mentioned that the use of the zonulin inhibitor, larazotide acetate, in a case series of four children with MIS resulted in a faster resolution of gastrointestinal symptoms and faster clearance time of the SARS-CoV-2 spike antigen (Yonker et al., 2022). This inhibitor is also being tested in a clinical trial (ClinicalTrials.gov identifier NCT05022303) in children with long-COVID, for whom a persistent inflammatory effect has been reported (Galán et al., 2022).

It should be noted that zonulin concentrations described in the studies available vary greatly. This variability could be explained by the shelf life of the samples and differences in the detection methods. Additionally, there are concerns regarding the validity of the ELISAs performed for zonulin quantification, in which the kits may detect not only zonulin but also zonulin-related proteins such as properdin (Scheffler et al., 2018). Another option is quantification of pre-haptoglobin 2 specifically (and no other zonulin-like proteins) with other methods such as western blot and/or mass spectrometry.

As mentioned before, a mounting body of evidence suggests that zonulin is a surrogate marker of tight junction permeability; however, estimation of microbial translocation could be reinforced using other known serum makers such as bacterial DNAemia, β-glucan, LPS-binding protein (LBP), intestinal-fatty acid binding protein (iFABP), soluble CD14 or others (Stehle et al., 2012; Koutsounas et al., 2015; Fukui, 2016), which could be options to consider alongside zonulin in future studies.

The present study has some limitations. The relatively small sample size for zonulin (n=60) and cytokine/chemokine values (n=42) might be responsible for type II errors, therefore some associations expected in previous studies, such as mortality and IL-6 values (Liu et al., in press), were found to be non-significant. This also contributed to the adjusted regressions; because of the small number of events (deaths), the regression models were adjusted for a few key confounders only to avoid model over-fitting. Thus, the findings presented herein are relevant, but a caveat must be made, as the magnitude of the associations should be interpreted with caution.

In conclusion, zonulin levels (as an indirect measure of microbial translocation levels) were detected in hospitalised patients with COVID-19 and were associated with death even after adjustment for age, gender, and obesity in a peruvian cohort of COVID-19. Future longitudinal studies on zonulin levels are recommended to determine their variation over time, as well as to identify their role in patients presenting systemic inflammatory syndrome/long-COVID.

## Data availability statement

The raw data supporting the conclusions of this article will be made available by the authors, without undue reservation.

## Ethics statement

The studies involving human participants were reviewed and approved by CIEI Universidad Cientifica del Sur-151-2022-POS50. Written informed consent was not provided because the samples were for diagnostic purposes, and, due to the first wave of COVID in Peru, the ethics committee approved the use of diagnostic samples.

## Author contributions

MP, LP-K and JR conceived the study. LP-K, MP, BY, AM performed zonulin quantification by ELISA. MU-G obtained the samples and clinical information. LP-K performed the statistical analyses. LP-K and MP wrote the manuscript. All authors made contributions to the draft and approved the final version. All authors contributed to the article and approved the submitted version.

## Funding

The present study was funded by *Beca Cabieses* from *Universidad Científica del Sur*.

## Acknowledgments

We would like to thank the personnel of the research laboratories of the *Universidad Científica del Sur* for their kind support.

## Conflict of interest

The authors declare that the research was conducted in the absence of any commercial or financial relationships that could be construed as a potential conflict of interest.

## Publisher’s note

All claims expressed in this article are solely those of the authors and do not necessarily represent those of their affiliated organizations, or those of the publisher, the editors and the reviewers. Any product that may be evaluated in this article, or claim that may be made by its manufacturer, is not guaranteed or endorsed by the publisher.
