# Corrigendum: Zonulin, a marker of gut permeability, is associated with mortality in a cohort of hospitalised peruvian COVID-19 patients

**DOI:** 10.3389/fcimb.2022.1062174

**Published:** 2022-12-15

**Authors:** Luciano A. Palomino-Kobayashi, Barbara Ymaña, Joaquim Ruiz, Ana Mayanga-Herrera, Manuel F. Ugarte-Gil, Maria J. Pons

**Affiliations:** ^1^ Grupo Enfermedades Infecciosas Emergentes. Universidad Científica del Sur, Lima, Peru; ^2^ Laboratorio de Cultivo Celular e Inmunología, Universidad Científica del Sur, Lima, Peru; ^3^ Grupo Peruano de Estudio de Enfermedades Autoinmunes Sistémicas, Universidad Científica del Sur, Lima, Peru; ^4^ Hospital Nacional Guillermo Almenara Irigoyen, EsSalud, Lima, Peru

**Keywords:** microbial translocation, zonulin, COVID-19, biomarker, ELISA, Peru

In the published article, there was a mistake in the Affiliations. Author Maria J Pons was affiliated with Affiliation 4 rather than Affiliation 1. The correct affiliations appear below. The authors apologize for this mistake.

Version of published affiliations:

*Luciano A Palomino-Kobayashi 1, Barbara Ymaña 1, Joaquim Ruiz 1, Ana Mayanga-Herrera 2, Manuel F Ugarte-Gil 3 4, Maria J Pons 4*

*1Grupo Enfermedades Infecciosas Emergentes. Universidad Científica del Sur, Lima, Peru.*

*2Laboratorio de Cultivo Celular e Inmunología, Universidad Científica del Sur, Lima, Peru.*

*3Grupo Peruano de Estudio de Enfermedades Autoinmunes Sistémicas, Universidad Científica del Sur, Lima, Peru.*

*4Hospital Nacional Guillermo Almenara Irigoyen, EsSalud, Lima, Peru.*

Correct version of the affiliation:

*Luciano A Palomino-Kobayashi 1, Barbara Ymaña 1, Joaquim Ruiz 1, Ana Mayanga-Herrera 2, Manuel F Ugarte-Gil 3 4, Maria J Pons 1*

*1Grupo Enfermedades Infecciosas Emergentes. Universidad Científica del Sur, Lima, Peru.*

*2Laboratorio de Cultivo Celular e Inmunología, Universidad Científica del Sur, Lima, Peru.*

*3Grupo Peruano de Estudio de Enfermedades Autoinmunes Sistémicas, Universidad Científica del Sur, Lima, Peru.*

*4Hospital Nacional Guillermo Almenara Irigoyen, EsSalud, Lima, Peru.*

